# Maternal Stress Induces Epigenetic Signatures of Psychiatric and Neurological Diseases in the Offspring

**DOI:** 10.1371/journal.pone.0056967

**Published:** 2013-02-22

**Authors:** Fabiola C. R. Zucchi, Youli Yao, Isaac D. Ward, Yaroslav Ilnytskyy, David M. Olson, Karen Benzies, Igor Kovalchuk, Olga Kovalchuk, Gerlinde A. S. Metz

**Affiliations:** 1 Department of Neuroscience, University of Lethbridge, Lethbridge, Alberta, Canada; 2 Department of Biological Sciences, University of Mato Grosso State, Caceres, Mato Grosso, Brazil; 3 Department of Biological Sciences, University of Lethbridge, Lethbridge, Alberta, Canada; 4 Departments of Obstetrics and Gynecology, Pediatrics and Physiology, University of Alberta, Edmonton, Alberta, Canada; 5 Faculty of Nursing, University of Calgary, Calgary, Alberta, Canada; Alexander Flemming Biomedical Sciences Research Center, Greece

## Abstract

The gestational state is a period of particular vulnerability to diseases that affect maternal and fetal health. Stress during gestation may represent a powerful influence on maternal mental health and offspring brain plasticity and development. Here we show that the fetal transcriptome, through microRNA (miRNA) regulation, responds to prenatal stress in association with epigenetic signatures of psychiatric and neurological diseases. Pregnant Long-Evans rats were assigned to stress from gestational days 12 to 18 while others served as handled controls. Gestational stress in the dam disrupted parturient maternal behaviour and was accompanied by characteristic brain miRNA profiles in the mother and her offspring, and altered transcriptomic brain profiles in the offspring. In the offspring brains, prenatal stress upregulated miR-103, which is involved in brain pathologies, and downregulated its potential gene target *Ptplb*. Prenatal stress downregulated miR-145, a marker of multiple sclerosis in humans. Prenatal stress also upregulated miR-323 and miR-98, which may alter inflammatory responses in the brain. Furthermore, prenatal stress upregulated miR-219, which targets the gene *Dazap1*. Both miR-219 and *Dazap1* are putative markers of schizophrenia and bipolar affective disorder in humans. Offspring transcriptomic changes included genes related to development, axonal guidance and neuropathology. These findings indicate that prenatal stress modifies epigenetic signatures linked to disease during critical periods of fetal brain development. These observations provide a new mechanistic association between environmental and genetic risk factors in psychiatric and neurological disease.

## Introduction

The gestational state is a period of particular vulnerability for both the mother and her offspring. Experience of distress during pregnancy may critically determine maternal health and alter offspring brain physiology and behaviour with life-long consequences [1], [2]. Gestational stress disrupts post-partum maternal care, which impedes brain and behavioural development of the offspring [3], [4]. It was proposed that the effects of maternal care are possibly transmitted across generations through non-genomic mechanisms [3]. Mechanisms of transfer include altered gestational endocrine milieu, maternal behaviour and transgenerational epigenetic programming [5]–[9]. Moreover, gestational stress directly influences fetal brain development and programming of hypothalamic-pituitary-adrenal (HPA) axis function [10], [11] to induce life-long changes in stress responsiveness [12] and possibly enhanced vulnerability to psychiatric conditions, including depression and bipolar affective disorder [13]–[16] and schizophrenia [17]–[20]. The prefrontal cortex in particular is relevant to mental health disorders, which may be precipitated or exaggerated by stress, pregnancy and childbirth [21]–[23].

Behavioural and physiological changes in stressed mothers and their offspring may be linked to altered gene expression in the brain, which is epigenetically regulated by experience. Epigenetic changes, including expression of microRNA (miRNA) enable rapid adjustments in gene expression without altering nucleotide sequences. Altered miRNA expression was suggested to prime neuroplasticity and physiological processes in response to early environment [8], [24] and the experience of stress [7], [25]. miRNA may be a critical component to mediate the effects of prenatal stress and maternal care on offspring development [26], [27]. Notably, miRNA expression is altered in many common psychiatric and neurological disorders, such as bipolar disorder, schizophrenia, autism, depression, and inflammatory conditions [28]–[33]. Most of these conditions share a suspected etiology that includes both the influence of adverse perinatal origins as well as a transcriptomic component, suggesting that epigenetic regulation of gene expression may represent a central common feature in individual disease etiology [34].

Here we provide a link between gestational adverse experience and epigenetic re-programming of the transcriptome by means of miRNA in the brains of gravid dams and their offspring. Maternal stress altered maternal antepartum behaviour and brain miRNA expression patterns in the frontal cortex, a region involved in maternal care, decision-making and stress responses. These changes translated to altered offspring miRNA signatures related to disease. Our observations allow proposing a mechanism by which gestational experience modulates gene expression with possibly life-long phenotypical consequences in the offspring.

## Materials and Methods

### 1. Experimental Design

Female rats stressed during late gestation and their non-stressed pregnant counterparts [*Stress* (n = 9) vs. *Non-stress* (n = 6) groups] were analyzed regarding their antepartum behaviour. Three additional dams per *Stress* and *Non-stress* groups were sacrificed the day of parturition (1 to 5 hours after delivery) and the frontal cortex was dissected for analysis of the microRNAome (miRNAome). One male pup from each of these six dams was used for miRNA expression analysis (n = 3 for each *Prenatal stress* and *Non-stress* groups). This study focused on frontal cortex of dams, due to its correlation with cognitive and stress related traits, and whole brains of male newborn offspring. To investigate epigenetic effects of maternal stress on the offspring, brains of male prenatally stressed (*Prenatal stress* group) and non-stressed (*Non-stress* group) newborn rats were collected for analysis of miRNAome and transcriptome.

### 2. Animals

Twenty-one timed-pregnant nulliparous female Long-Evans rats, bred and raised at the local vivarium, were used. Females were paired with a male for one hour per day until mating occurred. Pregnancy of the rats was confirmed by weight gain eleven days later. Pregnant rats were housed individually from gestational day 19 until delivery and recorded by an infrared video surveillance system (CCTV Cameras, Panasonic, USA).

### 3. Ethics Statement

All procedures were performed in accordance with the guidelines of the Canadian Council for Animal Care and approved by the University of Lethbridge Animal Welfare Committee (#0803).

### 4. Stress Procedures

#### Gestational

Timed-pregnant rats were stressed twice daily from gestational day 12 to day 18. Two stressors, restraint of the body for 20 min [35]–[37] and forced swimming in water at room temperature for 5 min [38]–[39] were applied daily. Restraint occurred in the morning and forced swimming in the afternoon hours.

### 5. Analysis of Antepartum Maternal Behaviour

Maternal behaviour was scored in gravid dams from 19–18 hours prior to delivery of the first pup. Tail chasing behaviour in the dams was scored as an indicator of maternal preparatory activity and care [40], [9]. The amount of time spent engaged in chasing (seconds) and manipulating the tail and the total number of rotations were measured as described previously [9].

### 6. Tissue Collection

*Brain.* Between 1 to 5 hours after parturition, dams and their offspring received a lethal dose of pentobarbital (Euthansol 100 mg/kg; CDMV Inc., Québec, Canada). Rats were rapidly decapitated and frontal cortex of mothers and whole brains of newborns were dissected and flash-frozen for mRNA and miRNA analysis.

### 7. miRNA and mRNA Expression Analysis

#### 7.1. RNA extraction

Total RNA was extracted from dams and newborn rat brains using TRI Reagent Solution (Applied Biosystems, Foster City, CA) according to the manufacturer’s protocol.

Samples from *Stress* dams and from *Prenatal stress* newborn rats were compared with non-stressed controls (dams and newborns from *Non-stress* group) for investigation of the effects of gestational stress in dams, and prenatal stress in newborns on brain miRNAome and transcriptome.

#### 7.2. miRNA microarrays

miRNA expression was analyzed using microarray technology performed by LC Sciences (Houston, TX) as described previously [41], [42]. The data were analyzed by first subtracting the background and then normalizing the signals using a LOWESS filter (Locally-weighted Regression) [43]. The putative gene targets for miRNAs differentially expressed by stress treatment were searched by computational analysis (TargetScan, Whitehead Institute for Biomedical Research, MIT, Cambridge, MA), which provided a list of predicted gene targets and related biological processes.

#### 7.3. Quantitative real time PCR (qRT-PCR)

In order to validate miRNAs modulated by gestational stress in dams, and prenatal stress in newborns determined by microarrays, we performed qRT-PCR analysis of eight differentially regulated miRNAs [44]. The same samples used for microarray analyses were also used for qRT-PCR validation (n = 3 per group, three replicates per sample). The following miRNAs were analyzed (5′ to 3′): mirR-181 and miR-186 (dams); miR-103, miR-151, miR-323, miR-145, miR-425, miR-98 (newborns). U6 snRNA was used as a reference control for calculation of the expression ratio. The generation of cDNAs from the total RNA samples was performed using M-MuLV Reverse Transcriptase, NEB#M0253S (New England Biolab, Ipswich, MA; see Table 1 for RT primers). qRT-PCR reactions were conducted with Bio-Rad CFX96™ Real-Time PCR Systems, using SsoFast™ EvaGreen® Supermix (Bio-Rad, Mississauga, ON) reaction premix added to the cDNAs templates and specific primers, according to the manufacturer’s protocol (see Table 1 for primer reference). A total volume of 12 µl was used, with 2.5 µl of cDNA template, 400 nM forward primer, 400 nM reverse primer, and 6 µl of SsoFast™ EvaGreen® Supermix (Bio-Rad, Mississauga, ON). Optimal dilutions and temperatures were adapted for each miRNA qRT-PCR reaction.

**Table 1:** Primers for qRT-PCR miRNA validation.

miRNA	Reverse Transcription Primer	Forward-primer	Reverse-primer
181	CACGGAACCCCGCCGACCGTGACCCAC	CCGCCGAACATTCATTGC	GACCGTGACCCACCGAC
186	GCTCAGACAGAAGTCACACTGAGCAGCCCA	CCCGCCGCAAAGAATTCTC	TCACACTGAGCAGCCCAAAAG
103	CACCGTTCCCCGCCGTCGGTGTCATAGC	CCCGCCAAGCCCTTACC	GCCGTCGGTGATGCTTTTTTGG
151	CACCGTTCCCCGCCGTCGGTGACTAGA	CCGCCTCGAGGAGCTCA	CCGTCGGTGACTAGACTGT
323	CACCGTTCCCCGCCGTCGGTGAGAGGT	CCCGCCCACATTACACGG	CCGTCGGTGAGAGGTCGA
145	CACCGTTCCCCGCCGTCGGTGAGGGAT	CCCGCCGTCCAGTTTTCC	CCGTCGGTGAGGGATTCCT
425	GACCGTTCCCCGCCGTCGGTCTCAACG	GGGCGAATGACACGATCAC	CGTCGGTCTCAACGGGAG
98	GCTCAGACAGAAGTCACACTGAGCAACAAT	CCGCGCGTGAGGTAGTAA	CCGTCACACTGAGCAACAATACAA
U6 snRNA (control)	CACCGTTCCCCGCCGUCGGTGCTTCTC	TGCTTCGGCAGCACATATAC	AGGGGCCATGCTAATCTTCT

#### 7.4. Gene microarray expression analysis

Prenatal stress effects on global gene expression were assessed by microarray technology. Samples used for miRNAome analyses were also used for transcriptome investigation (n = 3 per group). Total RNA was purified using the RNeasy total RNA clean up protocol (Qiagen, Manchester, UK). RNA samples were tested using Bioanalyzer Eukaryote Total RNA Nano Chip (Agilent, Mississauga, ON). The microarray protocol used here allows the simultaneous analysis of global mRNA expression profiles. Microarray analyses (probe synthesis, hybridization, and scanning) was performed using a standard Illumina platform protocol [45].

### 8. Statistical Analyses

Statistical analyses of maternal behaviour were performed using Statview software version 5.0 (SAS Institute, 1998). Behavioural data were standardized by square root transformation to fit a Gaussian curve histogram of normal distribution. Analysis of variance (ANOVA) and unpaired student t-tests were used for between-group comparisons. A p-value of less than 0.05 was chosen as significance level. All data are presented as mean ± standard error of the mean (SEM). Statistical analysis of miRNA and mRNA microarray data was performed using t-test between groups. T-values were calculated for each miRNA or mRNA, with p-values below a critical p-value (0.01) selected for cluster analysis. The clustering analyses used a hierarchical method and average linkage and Euclidean distance metric [46]. The relative miRNA levels were quantified using Bio Rad CFX Manager in the validation qRT-PCR.

## Results

### Gestational Stress Disrupts Antepartum Maternal Behaviour Along With miRNA Profiles

**Figure 1:**
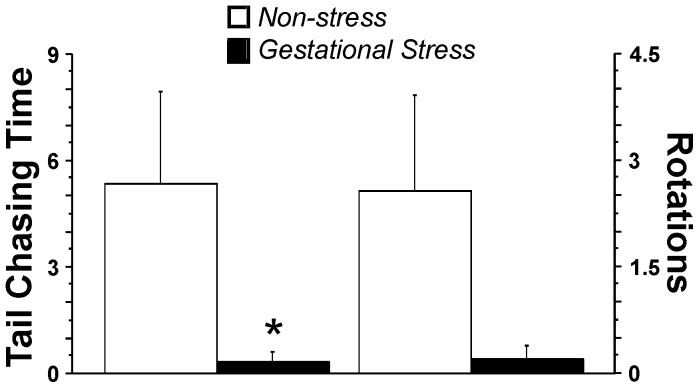
Gestational stress disrupts antepartum maternal behaviour. Time spent engaged in tail chasing behaviours and the number of rotations performed at 19–18 hours prior to delivery (all data transformed to square root). Gestational stress decreased the time spent in tail chasing activities and the number of rotations, indicating reduced maternal preparatory activity (n = 6 non-stress controls, n = 9 gestational stress). *p≤0.05, mean ± SEM.

Antepartum maternal tail chasing behaviour was scored frame-by-frame from cage-site videotapes. During the observation period, *Stress* dams spent significantly less time than *Non-stress* dams engaged in tail chasing behaviours, such as horizontal rotations (F(1,13) = 5.35, p<0.05; Figure 1). Furthermore, gestational stress reduced the number of rotations, although to a non-significant degree (F(1,13) = 4.43, p = 0.055).

**Figure 2:**
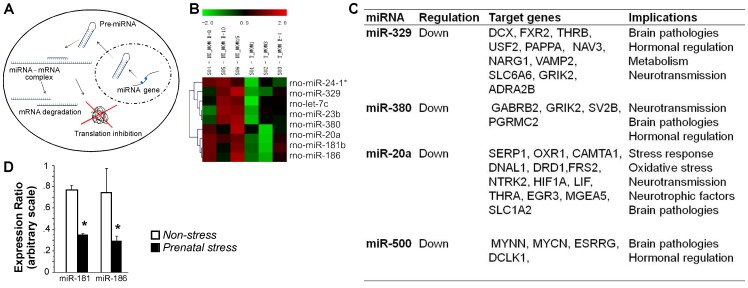
Gestational stress induces differential miRNA expression in frontal cortex. A, Schematic overview of miRNA biogenesis pathways. **B,** Heat map representation of differentially regulated miRNAs, as observed by microarray analysis. **C,** Table of target genes for miRNAs modulated by gestational stress (miR-329, miR-380, miR-20a, and miR-500; p≤0.05), and their physiological implications. **D,** Expression ratio group averages of miRNAs as observed by qRT-PCR analysis (p≤0.05). Note that prenatal stress downregulated miR-181 and miR-186 expression in the frontal cortex. miRNA analyses were performed in dams that showed representative behavioural characteristics (n = 3 per group, three repeats per sample). All data are presented as mean ± SEM.

Antepartum stress-induced behavioural alterations were accompanied by altered miRNA expression in the frontal cortex of dams. Since miRNAs in animals primarily inhibit translation of target mRNAs, decreases in miRNA levels should result in increased mRNA translation while increases in miRNA levels result in inhibition of translation (Figure 2A). A total of 342 miRNAs were differentially expressed in response to gestational stress (*Stress* vs. *Non-stress* groups). Overall, 195 miRNAs were downregulated and 147 miRNAs were upregulated. Gestational stress downregulated abundance of miR-329, miR-380, miR-20a, and miR-500 (all p≤0.05; Figure 2B-C). Stress also led to critical decreases in let-7c, miR-23b, miR-181, and miR186 amounts. Conversely, stress upregulated miR-24-1. The putative gene targets for these miRNAs were related to neuropathologies, neurotransmission, hormonal regulation, neurotrophic factors, stress response, oxidative stress and metabolism (Figure 2C). miR-181 and miR-186 were chosen for verification using qRT-PCR analysis. Downregulation of both miRNAs by gestational stress was confirmed (Figure 2D).

### Prenatal Stress Modulates Brain miRNAome and Transcriptome in Newborn Rats

**Figure 3:**
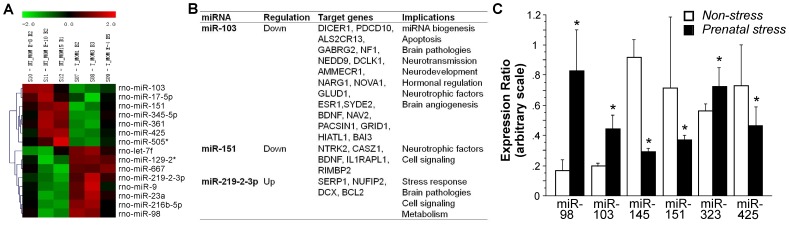
Prenatal stress modulates the brain miRNAome in male newborn offspring. A, Heat map representation of differentially regulated miRNA as observed by microarray analyses. **B,** Table of putative target genes for modulated miRNAs (miR-103, miR-151, and miR-219-2-3p; p≤0.05) and their physiological functions. **C,** Expression ratio group averages of miRNAs as observed by qRT-PCR analysis (p≤0.05). Whole brains of newborns born to dams shown in Figures 1 and 2 (n = 3 per group, three repeats per sample; 1 pup per dam) were used. All data are presented as mean ± SEM.

Analysis of the newborn brain miRNAome (*Prenatal stress* Vs. *Non-stress* groups Figure 3) shows a total of 336 miRNAs differentially expressed in response to prenatal stress, including 131 miRNAs whose abundance was downregulated and 205 miRNAs that were upregulated. The miRNAs differentially regulated by prenatal stress includes miR-23a (up), miR-129-2 (up), miR-361 (down), let-7f (up), miR-17-5p (down), miR-98 (up), miR-425 (down), miR-345-5p (down), miR-9 (up), miR216-5p (up), miR-667 (up), and miR-505 (down) (Figure 3A). Moreover, significant changes in expression due to prenatal stress were found in miR-103 (down), miR-151 (down), and miR-219-2-3p (up). The putative gene targets for these miRNAs includes genes related to miRNA biogenesis, apoptosis, brain pathologies, neurotransmission, neurodevelopment, hormonal regulation, neurotrophic factors, brain angiogenesis, cell signaling, stress response, and metabolism (Figure 3B).

From the miRNAs regulated by prenatal stress (*Stress* Vs. *Non-stress* groups), as observed by microarray analyses, the following candidates were selected for verification by qRT-PCR analysis: miR-151, miR-145, miR-425 (all down) and miR-103, miR-323, miR-98 (up) (Figure 3C).

**Figure 4:**
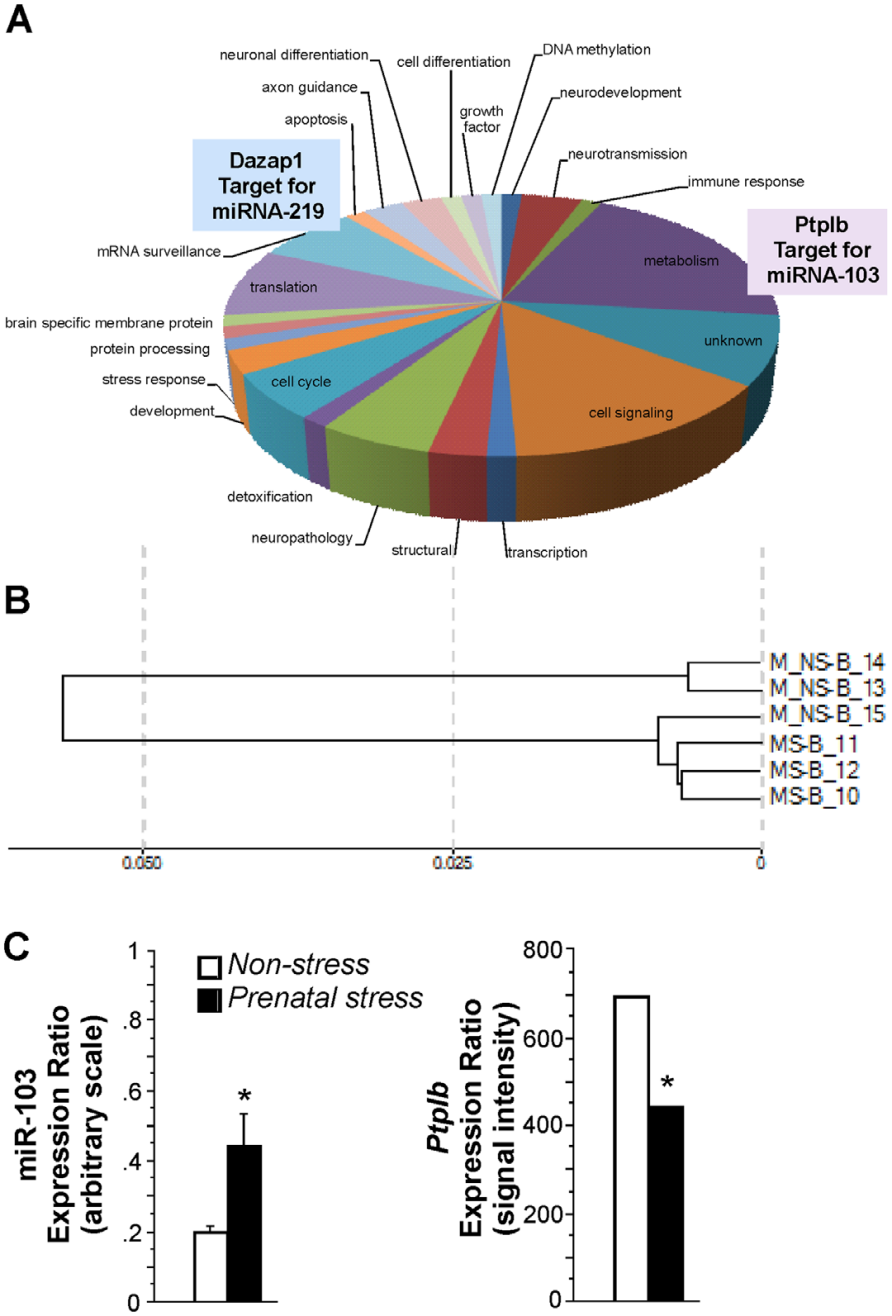
Prenatal stress alters the brain transcriptome in male newborn offspring. **A,** Differential global gene expression in the brains of prenatally stressed newborn rats. *Ptplb* and *Dazap1* are targets for miR-103 and miR-219, respectively. **B,** Clustering analysis of gene expression showed clusters of stressed and non-stress animals, except for one non-stressed animal. **C,** Prenatal stress elevated expression of miR-103, which coincides downregulation of its potential target *Ptplb* (mean ± SEM). Whole brains of newborns born to dams shown in Figures 1 and 2 were analysed (n = 3 per group, three repeats per sample; 1 pup per dam).

Global gene expression analysis revealed that 39 genes were downregulated by prenatal stress in the brains of newborn rats (more than 2 fold change; *Abhd14a, Argbp2, Cd47, RGD1559704, LOC310926, Klf10, Nsmce2, RGD1309216, Gramd1b, Itpr1, Tst, Pfkm, Vps11, Echs1, Zswim5, RGD1309388, Tmem176b, Cib1, Sfxn5, Cln8, Gucy1b3, Flii, Txnl4b, Ldha, RGD1561179, Zfp216, Ptplb, Galntl4, Pdia5, Herc1, RGD1305557, RGD1303003, RGD1305514, Aph1a, Visa, Clpb, RGD1563963, Snx1, Gstm1*) and 47 genes were upregulated (more than 2 fold change; *P4hb, RGD1560212, RGD735065, LOC498346, Rps3a, LOC497732, Wbp11, Taf9b, RGD1560975, Lpar1, Rnf7, LOC500829, Chp, LOC300760, Pgrmc1, LOC500398, LOC688712, Cd2bp2, RGD1561219, RGD1565840, RGD1560186, LOC497745, LOC497720, LOC500344, Mcts1, RGD1564956, LOC498644, Rala, Sfrs6, Mrlcb, Ptn, Sfrs5, Hdac2, LOC500533, LOC501553, Dazap1, Fem1b, RGD1563431, Cct4, Rbbp7, RGD1308165, Acsl4, Ppp1r14b, LOC498449, Usmg5, RGD1560729*). Biological processes affected by these genes include DNA methylation, neurodevelopment, neurotransmission, immune response, growth factor, cell differentiation, neuronal differentiation, axon guidance, apoptosis, mRNA surveillance, translation, brain specific membrane protein, protein processing, stress response, development, cell cycle, detoxification, neuropathology, structural maintenance, transcription, cell signaling and metabolism (Figure 4A). Clustering analysis of gene expression revealed clusters of animals from *Prenatal stress* and *Non-stress* groups, except for one animal from the *Non-stress* group (Figure 4B).

Among genes modulated by prenatal stress with function in metabolic processes, the gene *Ptplb* was downregulated. *Ptplb* is a putative target for miR-103, which was upregulated by prenatal stress (Figure 4C). Furthermore, *Dazap1* was upregulated by prenatal stress. *Dazap1* is a gene related to mRNA surveillance, i.e. regulation of gene expression, which is a putative target for miR-219.

## Discussion

The developmental origins of health and disease have become a current topic of interest. Although it is widely accepted that the perinatal period represents a stage of particular vulnerability for the developing brain, the causal mechanisms and long-term consequences of perinatal programming are poorly understood. Here we show that epigenetic regulation through miRNA represents a critical step in stress-induced gene expression and is accompanied by characteristic maternal behavioural traits and signature analogues of human psychiatric and neurological disease.

The developing brain is particularly vulnerable to adverse intrauterine conditions and responds to altered endocrine milieu with re-programming of the hypothalamic-pituitary-adrenal (HPA) axis and associated behavioural and physiological responses [47], [48]. These endocrine changes may have important implications for the vulnerability to mental disorders. Stress from gestational days 12 to 18 in rats corresponds to the second trimester of pregnancy in humans, which is thought to be the most sensitive period to influence offspring brain morphology [49] and determine mental health in later life [50], [51]. Our findings indicate that maternal stress may affect critical periods of fetal neurodevelopment through dynamic regulation of miRNA in both the mother and her offspring.

### Gestational Stress Disrupts Antepartum Maternal Behaviour Along with Epigenetic Re-programming

Antepartum maternal behaviour, such as tail chasing and rotational behaviours, may be reflective of preparatory activities. Preparatory activities include nest building, which increase during the last 24 hours preceding parturition [52]. Since a similar time course was found for tail chasing behaviour [9], the present findings suggest that preparatory activities are sensitive to maternal stress. The lack of activities observed in stressed dams may reflect a lack of motivation, a central component of depression-like behaviour linked to stressful experiences [53], [54]. If antepartum activities are somewhat predictive of postpartum maternal care [55], [26], [3], even a moderate behavioural change in maternal behaviour may potentially have significant consequences for offspring development.

Behavioural findings in stressed dams were accompanied by altered epigenetic profiles in the frontal cortex, including downregulation of miR-181b. The miR-181 family is particularly enriched in the brain and is involved in autism spectrum disorders [56], schizophrenia [57], Alzheimer disease [58], where they are mainly found to be upregulated. Downregulation of miR-181 contributes to accelerated HIV-associated dementia in opiate abusers [59]. At the cellular level, miR-181 regulates apoptosis factors such as bcl-2 in astrocytes. Downregulation of miR-181 was shown to have protective effects against apoptosis and mitochondrial dysfunction [60]. Gestational stress also downregulated miR-186 in the maternal frontal cortex, which is in contrast to the upregulation found in frontal cortex, hippocampus, and cerebellum in male rats [25]. The present findings do not allow drawing a causal relationship between the behavioural phenotype and epigenetic changes, however, altered miRNA expression in the maternal frontal cortex may have relevance to pregnancy-related mental and emotional changes in stressed mothers.

### Prenatal Stress Alters miRNA Signatures in the Offspring

Prenatal stress modified expression of genes that are central to brain development and plasticity, including apoptosis, neurotransmission, neurotrophic factors, and cell signaling. One particularly interesting finding is the upregulation of miR-103 and downregulation of its putative gene target *Ptplb* in brains. miR-103 is enriched in the cortex [61] and its expression increases during neurodevelopment, particularly cell differentiation [62], [63] and translation [64]. In the mature brain, however, upregulation of miR-103 may suppress BDNF synthesis in humans [65] and promote neuropathological processes in a mouse model of Alzheimer’s disease [66]. Accordingly, perinatal adversity may increase the risk of cognitive decline [67], [68] and elevate the vulnerability of cholinergic neurons [69]. Altered miR-103 expression in the developing brain may therefore contribute to cognitive changes in adulthood. The putative gene target of miR-103, *Ptplb*, is essential for biosynthesis of tyrosine phosphatase-like member b, which is involved in a wide range of neuronal functions, including synapse formation [70], disorders involving the frontal cortex such as Alzheimer’s disease [71], [72] and schizophrenia [73]. miR-103-mediated inhibition of *Ptplb* translation may contribute to alterations in behavioural and neuronal plasticity in prenatally stressed offspring.

Another duo, miR-219 and its putative gene target *Dazap1* were upregulated by prenatal stress in newborns, suggesting parallel regulatory interference in gene expression. Notably, miR-219 may be implicated in the pathology of schizophrenia and bipolar affective disorders [30], both of which are closely linked to prenatal stress [20], [18] and altered HPA axis activity [74], [14], [16]. miR-219 modulates excitatory synaptic plasticity through N-methyl-D-aspartate (NMDA) glutamate receptors [28], [75]. Disruption in NMDA receptor function through miR-219 regulation results in aberrant hyperlocomotor behaviour in mice [28]. Thus, stress through regulation of miR-219 may interfere with developmental neuronal plasticity and behaviour.

Further changes in miRNA profiles included miR-323, which modulates host-pathogen interactions, such as those involved in HIV-1 [76] and H1N1 Influenza A [31]. miR-323 binds to the PB1 virus gene and may assist in the defense against viral replication [31] and thus have protective functions against stress-induced vulnerability to pathogens [77], [78]. By contrast, recent evidence points towards miR-323 as a positive regulator of Wnt/cadherin signaling to upregulate pro-inflammatory mechanisms and potentiate cell migration, proliferation and adhesion in the pathogenesis of rheumatoid arthritis [79], [80]. On the other hand, prenatal stress also upregulated miR-98 expression, which modulates immune responses through cytokine pathways [81], and was shown to downregulate the production of the proinflammatory cytokine IL-10 in macrophages [82]. Both miR-323 and miR-98 upregulation in brains of prenatally stressed offspring may indicate an altered pro-inflammatory state in the brain. By contrast, it is generally assumed that prenatal stress increases the vulnerability to immune disorders [83], which may also apply to the brain [84]. However, in line with potentially protective effects of miR-323 upregulation, mouse studies have also found that maternal stress may enhance anti-viral immunity, for example by promoting the protection against herpes simplex virus [85], [86]. It is possible that these miRNA changes partially mediate a defensive response against acute infections in newborns.

Altered responses to immune challenges during early development were also suggested for the pathogenesis of multiple sclerosis (MS) [29]. While miR-145 has a regulatory role in embryonic neuronal differentiation in rats [87], it is also differentially expressed in MS-afflicted human patients, thus providing a potential epigenetic marker of this condition [29]. The current findings show that prenatal stress downregulates brain miR-145, as opposed to its upregulation in human blood cells in MS [29]. Since heredity represents a proposed risk factor for MS [88], early adverse experiences may translate environmental influences into epigenetic signatures to affect neuronal plasticity and the predisposition for neurological disease in later life [89], [90].

In spite of continuous epigenetic re-programming throughout a lifetime [91]–[93], early epigenetic imprints may persist into later life [94]–[96]. For instance, epigenetic modification in somatic cells may perpetuate throughout life by stable mitosis [5], [96]. The frontal cortex in particular may be relatively resistant to epigenetic re-programming by lifespan environmental influences compared to other brain areas, as indicated by human developmental cortex maps [97]. Thus, perinatal programming by persistent patterns in miRNA regulation may contribute to psychiatric and neurological conditions in later life.

### Integrating Maternal and Fetal Physiological and Epigenomic Features

The effects of prenatal stress have been well characterized with respect to critical periods in early development [98], [99], [90]. The nature and duration of maternal stress likely determine the physiological and epigenomic responses in the offspring, however, the gestational timing of the stressor may represent a particularly crucial influence on brain development and maturation [100]. It is not yet clear exactly how the maternal endocrine response to stress programs the epigenome of their offspring. It is known that excessive glucocorticoid levels can cross the protective enzymatic barrier of the placenta to reach the fetal brain [101]. Here, elevated levels of glucocorticoids may, through dynamic regulation of miRNA expression, alter the expression of critical genes involved in sexually dimorphic brain organization [90]. Furthermore, it has been recently shown that psychological stress in adulthood influences central miRNA expression [25]. These direct effects of stress on the brain mircoRNAome may at least in part contribute to an epigenomic imprint in the mother’s brain and contribute to cortical plasticity and neuromorphological remodeling that is characteristic for the post-partum brain [102].

### Conclusions

Here we provide evidence for a possible link between gestational adverse experience and epigenetic re-programming via altered miRNA expression in the brains of gravid dams and their offspring. Mild gestational stress disrupted behaviour in the parturient dam and altered miRNAs in the frontal cortex, a region involved in maternal care, decision-making, and stress responses, and epigenetic regulators of gene expression in the newborn offspring. The present findings propose a mechanism by which gestational experience modulates gene expression with possible phenotypic consequences. Because miRNAs have been recognized as important biomarkers of disease states in humans, their dynamic regulation by stress proposes a promising therapeutic avenue for intervention of disease predisposition in at-risk pregnancies.
